# GIS-Based Analysis for UAV-Supported Field Experiments Reveals Soybean Traits Associated With Rotational Benefit

**DOI:** 10.3389/fpls.2021.637694

**Published:** 2021-05-31

**Authors:** Yuya Fukano, Wei Guo, Naohiro Aoki, Shinjiro Ootsuka, Koji Noshita, Kei Uchida, Yoichiro Kato, Kazuhiro Sasaki, Shotaka Kamikawa, Hirofumi Kubota

**Affiliations:** ^1^Graduate School of Agricultural and Life Sciences, Institute for Sustainable Agro-Ecosystem Services, The University of Tokyo, Tokyo, Japan; ^2^Graduate School of Agricultural and Life Sciences, The University of Tokyo, Tokyo, Japan; ^3^Department of Biology, Kyushu University, Fukuoka, Japan; ^4^Plant Frontier Research Center, Kyushu University, Fukuoka, Japan; ^5^Japan Science and Technology Agency, PRESTO, Kawaguchi, Japan

**Keywords:** crop rotation, drone, experimental design, legume, wheat, yield

## Abstract

Recent advances in unmanned aerial vehicle (UAV) remote sensing and image analysis provide large amounts of plant canopy data, but there is no method to integrate the large imagery datasets with the much smaller manually collected datasets. A simple geographic information system (GIS)-based analysis for a UAV-supported field study (GAUSS) analytical framework was developed to integrate these datasets. It has three steps: developing a model for predicting sample values from UAV imagery, field gridding and trait value prediction, and statistical testing of predicted values. A field cultivation experiment was conducted to examine the effectiveness of the GAUSS framework, using a soybean–wheat crop rotation as the model system Fourteen soybean cultivars and subsequently a single wheat cultivar were grown in the same field. The crop rotation benefits of the soybeans for wheat yield were examined using GAUSS. Combining manually sampled data (*n* = 143) and pixel-based UAV imagery indices produced a large amount of high-spatial-resolution predicted wheat yields (*n* = 8,756). Significant differences were detected among soybean cultivars in their effects on wheat yield, and soybean plant traits were associated with the increases. This is the first reported study that links traits of legume plants with rotational benefits to the subsequent crop. Although some limitations and challenges remain, the GAUSS approach can be applied to many types of field-based plant experimentation, and has potential for extensive use in future studies.

## Introduction

Since R. A. Fisher’s initial work on statistical principles of experimental design (Fisher, 1926), field experimentation has played a pivotal role in variety of plant sciences, including ecology, evolutionary biology, forestry, and crop science (Box, 1980; Edmondson, 2005). In field experiments, measured values may vary among the experimental plots owing to the treatments, but there is always some degree of additional variation caused by both systematic errors, e.g., spatial variation in topography and soil fertility, and random errors, e.g., variations from sampling procedure (Sokal and Rohlf, 1995). Although many studies have addressed methods to minimize these errors, the results of field experiments are still subject to large unwanted and uncontrolled variability (Hurlbert, 1984; Legendre et al., 2004; Payne, 2006; Yang, 2010). In addition, a key factor in field experimentation is the time-consuming nature of sampling coupled with limited availability of time, resulting in small numbers of samples, i.e., small sample size (Hurlbert, 1984). Small sample size can cause significant analysis problems by reducing the statistical power and inferential confidence, especially for data with large systematic and random errors (Nakagawa and Cuthill, 2007). Therefore, the development of cost-effective, high-throughput, and general-purpose measurements and their analytical framework is needed to extend the effectiveness of field experimentation studies (Edmondson, 2005).

It is well known that legume plants such as soybeans can acquire nitrogen from the atmosphere through a mutualistic symbiosis with rhizobia, thereby providing a crucial service to the ecosystem. The use of legumes in crop rotations for their nitrogen-fixing ability has a long history (Chorley, 1981; Stinner et al., 1992); it is widely practiced in both industrialized and developing countries (Giller and Cadisch, 1995; Becker and Johnson, 1998; Biederbeck et al., 2005). Many research projects have quantified this nitrogen contribution and its net effect on subsequent crop yields (Herridge and Rose, 2000; van Kessel and Hartley, 2000; Walley et al., 2007; Anglade et al., 2015; Duc et al., 2015). Interestingly, those studies have found the effects of legume cultivation to be quite variable. Although many studies report positive effects of legume cultivation on subsequent crop yield, others have found neutral or even negative effects (reviewed in Walley et al., 2007; Anglade et al., 2015). Such variations can be partially explained by the extreme variability in nitrogen fixation among different legume crops and among the cultivars used for experimentation. Importantly, however, the large systematic and random errors associated with crop rotation experiments can increase the variability within results, and thereby reduce the statistical power of the data analyses. In addition, the requirement for two different crops to be grown sequentially in the same field in crop rotation trials can magnify the potential for errors during experiments.

To predict the effects of legume cultivation on subsequent crops, to maximize their benefit, and to develop innovative genotypes that can enhance rotational benefits, it is necessary to identify which legume traits are associated with the rotational benefits (Herridge and Rose, 2000). Comparison of rotational effects among cultivars of the same legume species may be the best way to achieve this goal, but no studies have verified differences in rotational benefits among cultivars (Duc et al., 2015). One reason for this lack of research may be the difficulty in detecting cultivar differences using conventional field experiments and statistical methods (e.g., ANOVA), because differences among cultivars are relatively small, whereas the variations in rotational experiments are large. To overcome this challenge, a method is needed to examine the differences in rotational benefits among legume cultivars, and to determine which legume traits are associated with rotational benefits.

Recent advances in technical devices and analytical methods have made it possible to do cost-effective remote sensing of field-cultivated plants (Houle et al., 2010; Furbank and Tester, 2011; Tardieu et al., 2017; Tripodi et al., 2018). Proximal sensing through the use of unmanned aerial vehicles (UAVs) is among the most promising and popular techniques, because it is rapid, non-destructive, cost-effective, and information dense (Sankaran et al., 2015; Yang et al., 2017; Guo et al., 2018; Maes and Steppe, 2019). UAV remote sensing enables the acquisition of a large amount of image data accompanied by location information. Recent studies have shown that UAV sensing and image analysis can be used to estimate several traits of field-cultivated plants, e.g., cover area, volume, height, and normalized difference vegetation index (NDVI) (Guo et al., 2017, 2020; Watanabe et al., 2017; Zhou et al., 2017; Hassan et al., 2019). These techniques allow researchers to cost-effectively and non-destructively obtain large amounts of pixel-level plant canopy data with location information. However, despite these significant benefits, there is no general methodology to integrate the large quantities of image data from UAV remote sensing with the manually collected data from conventional field experimentation.

**FIGURE 1:**
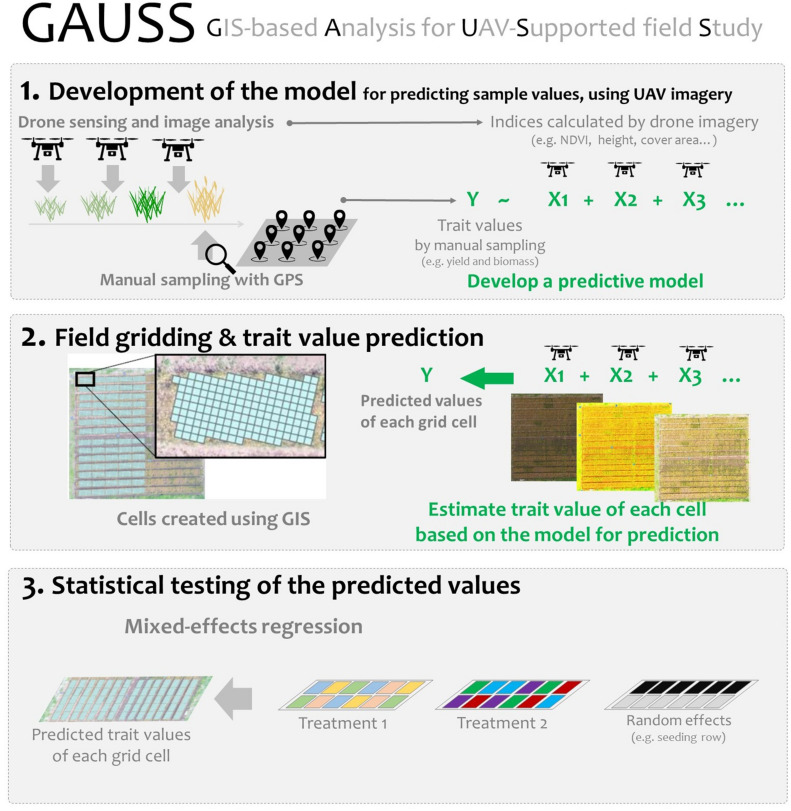
Steps in the GIS-based analysis for UAV-supported field study (GAUSS) framework.

To investigate these issues, we proposed a simple analytical framework, i.e., geographic information system (GIS)-based analysis for UAV supported field study (GAUSS), to integrate remote sensing data into a conventional field experiment (Figure 1). A field cultivation experiment was conducted to examine the effectiveness of the GAUSS framework, using a soybean–wheat [*Glycine max* (L.) Merr.; *Triticum aestivum* L.] crop rotation as the model system. The differences among soybean cultivars in their effect on wheat yield were examined, and the following questions were addressed:

(1) Which type or combination of indices from the UAV imagery best predicted wheat yields?
(2) How did the whole distribution of estimated yields in a plot differ from the actual yield data from manual sample collection at selected locations?
(3) Were there differences in wheat yields associated with different soybean cultivars?
(4) What traits of the soybean cultivars were associated with the wheat yields?

## Materials and Methods

### Field Cultivation of Soybean and Wheat

The study was conducted from June 2018 to June 2019 at the Institute of Sustainable Agro-ecosystem Services (ISAS), the University of Tokyo, Japan (35°43′N, 139°32′E). Soybeans were grown during the summer (June to October), followed by winter wheat in the winter (November to June). The soil was derived from a volcanic ash, classified as a Typic Melanudand (USDA Soil Taxonomy). Climatic data during the soybean and wheat growing seasons are summarized in Supplementary Table 1.

**FIGURE 2:**
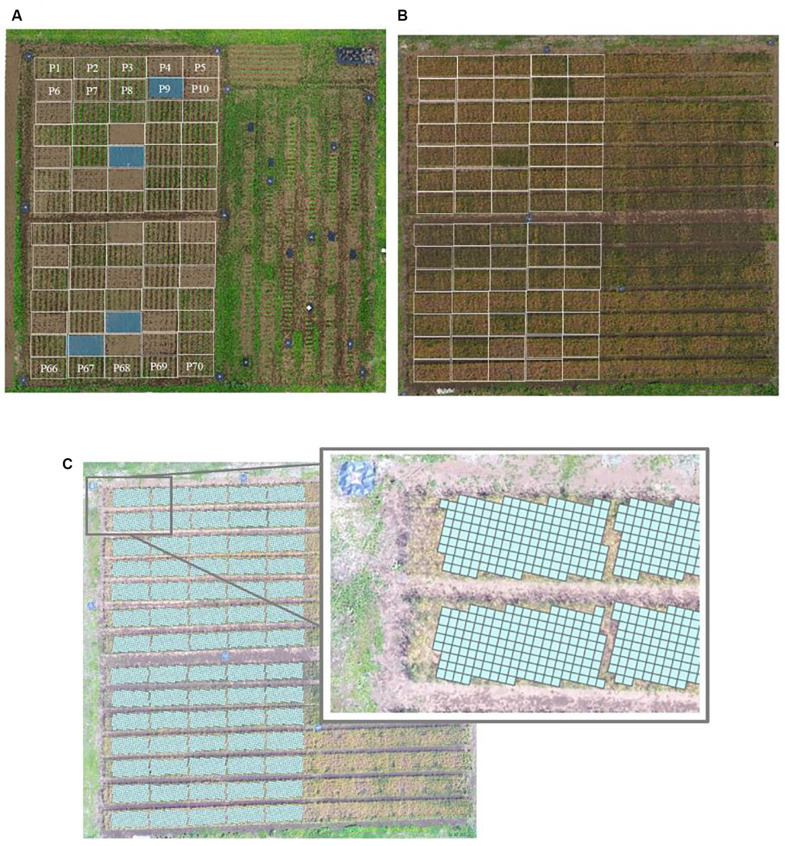
Experimental field P1–70 used in the work reported here. **(A)** Soybean (*Glycine max*) cultivation plots; **(B)** subsequent wheat (*Triticum aestivum*) cultivation, with locations of preceding soybean plots superimposed on the image; **(C)** the field divided into 25-cm × 25-cm cells, using the UAV aerial surveillance data.

Fourteen soybean cultivars (Supplementary Table 2) with different plant types and yield potentials were used (Kaga et al., 2011). Seeds of the GmWMC (Glycine max world mini core-collection) line were obtained from the current Genetic Resource Center, National Agriculture and Food Research Organization, Tsukuba, Japan. There were 70 experimental plots: 56 plots for soybean cultivar testing, 4 plots where the natural weed community was allowed to develop (weedy), 4 plots where the soil was covered with an anti-weed covering (sheet), and 6 plots for destructive sampling of soybean plants (Figure 2A and Supplementary Table 3). Each plot was 10.08 m^2^ (2.4 m × 4.2 m). The cultivars were assigned to the plots in a randomized design, with 2–6 replicates (Supplementary Table 3). Three soybean seeds were sown per hill, on 20 June or 20 July 2018, with a row spacing of 60 cm and a hill spacing of 30 cm. Hills were thinned to one seedling at 3 weeks after sowing. A basal fertilizer (N:P:K, 3:10:10) was applied at a rate of 1,000 kg ha^–1^ for soybean cultivation.

The above-ground parts of the soybean plants were manually harvested at physiological maturity during October 2018 and dried completely at 80°C. The whole-plant above-ground dry weight, stem dry weight, seed dry weight, and 100-seed weight were measured. The below-ground parts were left in the soil. The field was tilled twice to a depth of 15 cm using a rotovator at 2 weeks after the soybean harvest. In November 2018, wheat (“*Satonosora*”) was uniformly sown over the entire area (40 m × 50 m), including both the location of the 70 soybean plots and the adjacent field area (Figure 2B), at 80 kg ha^–1^. No fertilizer was applied for wheat cultivation. Standard crop protection practices for soybean (manual weeding, pesticide application, intertillage, and molding) and wheat (herbicide application, manual weeding, and fungicide application) were followed.

During 5 to 10 June 2019, the above-ground parts of the wheat plants were manually harvested from 154 sampling points (1 m × 1 m; Supplementary Figure 1) by cutting at the soil surface and placed in a mesh bag. The 154 sampling points comprised one each in the 70 soybean plots and 84 in the adjacent area of the field. A local area RTK-GPS (Real-Time Kinematic Global Positioning System) that was conducted with Hemisphere GNSS devices (Hemisphere GNSS, Scottsdale, AZ, United States) was used to determine the locations of the sampling points. After drying completely at 80°C, the harvested wheat samples were weighed and sorted into immature ears, mature ears, and straw. The dry weights of mature ears and straw were measured separately. The number of mature ears was counted.

### GAUSS: An Analytical Framework to Estimate Data Values From UAV Imagery

The GAUSS general framework was used to integrate remote sensing data into a conventional field experiment that investigated plant growth benefits in a soy–wheat crop rotation. The three main steps of this framework (represented in Figure 1) were:

1 Development of the model for predicting sample values, using UAV imagery
2 Field gridding and trait value prediction
3 Statistical testing of the predicted values

### Detailed GAUSS Methodologies

#### Development of the Model for Predicting Sample Values, Using UAV Imagery

##### Acquisition of Image Data

**FIGURE 3:**
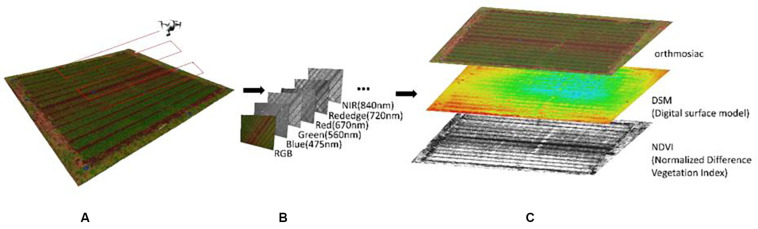
Representation of the image capture and data processing in the GAUSS system. **(A)** UAV overflight to capture image data; **(B)** types of image data collected; **(C)** results of image processing.

UAV remote sensing was carried out on 15 February, 14 March, and 12 April 2019, when the target wheat plants were in various stages of growth (tillering, early growth, and lager growth stage). A commercial-grade UAV (DJI Inspire 1, Shenzhen, China) equipped with a multispectral camera was used. The UAV was flown automatically over the field (Figure 3A) at an altitude of 30 m under the control of a commercially available flight application (Litchi, VC Technology Ltd., London, England). Two cameras—a Zenmuse X5 (DJI, Shenzhen, China) and a Micasense RedEdge (Micasense, Seattle, WA, United States)—were mounted on the UAV to ensure that RGB and multispectral images were captured during the same flight (Figure 3B). Two sets of images of a calibrated reflectance panel placed at about 1 m height were also captured immediately before and after each flight to improve the accuracy of the reflectance data for multi-spectral images. Also, acrylic plates were placed at the four corners of the field and three locations within the field as ground control points (GCPs), and were measured using the Hemisphere RTK differential GNSS device to improve the geolocation accuracy.

The captured images were processed using commercial photogrammetry software (Pix4Dmapper Pro, Pix4D, Lausanne, Switzerland). The pixel-by-pixel values over the entire wheat field were determined by using orthomosaic and digital surface modeling (DSM) that are generated from RGB images to calculate the vegetation cover area and plant height, respectively, and reflectance maps were generated from the multi-spectral images to calculate the NDVI (Figure 3C).

##### Developing the Model for Predicting Wheat Yield

To determine which of the image indices best predicted actual wheat yield, the model selection based on the Akaike’s information criteria (AIC) (Akaike, 1974) for results of generalized linear models (GLMs) was used. Accurate geolocation information for both the UAV imagery and the manual sampling points made it possible to map the manually sampled data (actual ground data) into the UAV imagery indices. In the GLM analysis, the dry weight of the harvested wheat ears from each manual sampling location was the response variable. The means of vegetation cover area, plant height, and NDVI at each of the sampling locations, estimated from the UAV images recorded in February, March, and April, were the explanatory variables. Plant height from February was excluded from the analysis owing to low plant height (<10 cm) and consequent low estimation accuracy. The error distribution was Gaussian with an identity link function. The statistical model with the lowest AIC score was selected as the best model (*f*) for estimating the values of the manually sampled wheat yield data (*y*_**p**_) at a sampling point **p** from the UAV images, where:

(1) $${\hat{y}}_{p}=f\,\left(x_{1,p},x_{2,p},\cdots ,x_{m,p}\right)+\varepsilon$$

and ${\hat{y}}_{p}$ is the estimate of *y*_**p**_, *x*_*i*,**p**_ represents *i*-th index (vegetation cover area, plant height, or NDVI in this study) at a sampling point **p** derived from the UAV images, *m* is the total number of indices, and ϵ is measurement error, respectively. The best model was then used in all subsequent steps.

#### Field Gridding and Trait Value Prediction

With the best model, the wheat yield over the entire field was calculated in ArcGIS Spatial Analyst v. 10.6 software (Esri, Redlands, California, United States) to map the aerial photographs. The field was divided into 25 × 25 cm cells (*c*_1_, *c*_2_, ⋯, *c_n_c__*, where *n*_*c*_ is the total number of cells) using GIS. With the model (*f*), the pixel-by-pixel predicted values of the target traits, including wheat yield, were calculated for the entire field (${\hat{y}}_{p_{1}},{\hat{y}}_{p_{2}},\cdots ,{\hat{y}}_{p_{n_{\text{p}}}}$, where *p_i_* is the *i*-th pixel in the UAV image of the field, and *n*_*p*_ is the total number of pixels in the image). Then the average yield of wheat in each cell (*c*_*i*_) was calculated from the pixel-by-pixel values:

(2) $${\hat{y}}_{c_{i}}=\sum_{p\,\text{in}\,c_{i}}{\hat{y}}_{p}/n_{c_{i}}$$

where *n*_*c_i_*_ is the total number of pixel in each cell.

Cells that contained two plots during soybean cultivation and cells containing both corridor and experimental plot areas during wheat cultivation were eliminated (Figure 2C). This resulted in 8756 cells of predicted wheat yield values (mean of 125 cells per soybean plot). To assess the relationship between predicted yield and manually sampled yield, the overall distributions of these values for each plot were compared.

#### Statistical Testing of the Predicted Values

The experimental factors affecting the predicted wheat yields of each cell were analyzed in two ways. The first set of analyses examined whether the spatial variation of wheat yield was affected by the previously grown soybean cultivar, using a generalized linear mixed model (GLMM) with Gaussian distribution and identity link. In this model, predicted wheat yields in each cell were treated as the response variable, and sowing date and cultivar were treated as the explanatory variables. To account for systematic error due to spatial variations in the field and to avoid pseudo-replication caused by repeated observations from the same plots, the sowing row and plot identity (nested within sowing row) were treated as random effects. A significant difference among cultivars was found, so pairwise comparisons between the cultivars that produced the lowest wheat yield and other cultivars were carried out.

The second set of analyses investigated which soybean traits affected the yield of the subsequently grown wheat, again using the GLMM with Gaussian distribution and identity link methodology. Predicted wheat yields of each cell were treated as the response variable, and soybean stem dry weight, seed dry weight, above-ground dry weight, 100-seed weight, and their first-order interactions were treated as explanatory variables. These are typical soybean traits that are measured (Cui et al., 2001; Kaga et al., 2011; Qiu et al., 2013). The sowing row and plot identity (nested within sowing row) were treated as random effects.

The “lme4” package and the “lmer” function in the R software environment for GLMM analyses (R Development Core Team, 2010; Bates et al., 2014) were used. The likelihood ratio test was used to test the significance of the GLMM results. Graphs of GLMM predictions were drawn in the sjPLot package for R (Lüdecke, 2018).

## Results

### Model Selection for Wheat Yield

**FIGURE 4:**
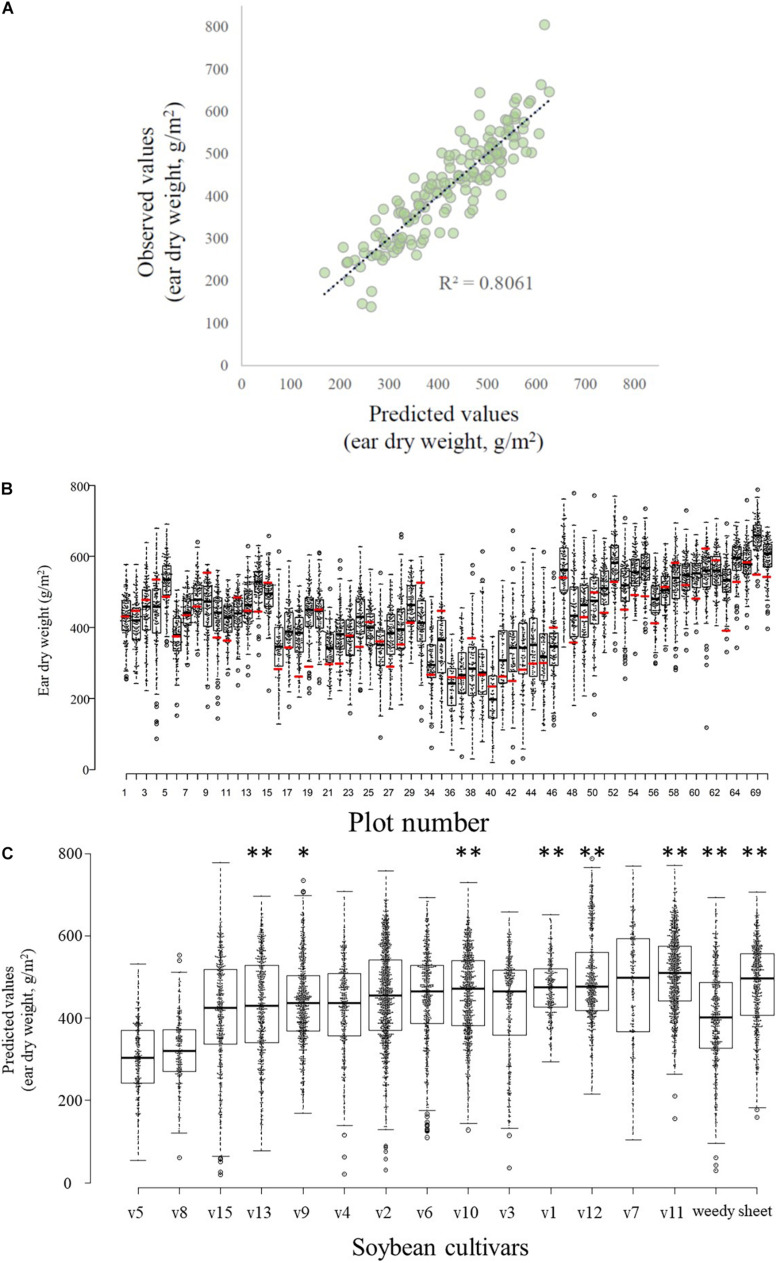
Relationships between predicted and observed values. **(A)** Yields (wheat ear dry weight) predicted by the best model vs. observed values. **(B)** Boxplots and scatter plots of the distribution of predicted wheat yields in each experimental plot (black bar within a box indicates median predicted yield; box bottom and top, 25 and 75% quartiles, respectively, whiskers, 1.5× the interquartile range; open circles outside the box, outliers) and manually sampled yield values (red bar). Note that the 6 field plots (P31–33, P66–68) used for destructive sampling of soybean (*Glycine max*) plants are not included here. A total of 64 box plots are shown, therefore the numbering of the *x*-axis is discontinuous. **(C)** Effects of the different soybean cultivars grown before the wheat crop on the predicted wheat ear dry weights. Boxplot features are as described in **(B)**. Asterisks indicate significant differences (^∗^*P* < 0.05; ^∗∗^*P* < 0.01) between the predicted values for cultivar v5 (which produced the lowest wheat yield) and each other soybean cultivar or weed management method.

AIC showed that the best model included four UAV-based indices (i.e., vegetation cover area on 14 March and 12 April, height on 14 March, and NDVI on 12 April; Table 1). There was a high correlation (*R*^2^ = 0.8061) between the values predicted by this model and the observed manually measured yield (Figure 4A). Among the models that were explored, all of the top 20 included both cover area on 14 March and NDVI on 12 April (e.g., Table 1).

**TABLE 1:** The results of model selection, ranked by Akaike information criteria (AIC), in the search to identify the best model for predicting ear dry weight of wheat (*Triticum aestivum*) from UAV imagery data.

Model No.	Explanatory variables included in the models							*R*^2^	DF	AIC	ΔAIC	Weight
1		C_Mar.14	H_Mar.14		C_Apr.12		N_Apr.12	0.8061	6	2375.4	0	0.082
2		C_Mar.14			C_Apr.12		N_Apr.12	0.8035	5	2375.5	0.03	0.081
3		C_Mar.14	H_Mar.14		C_Apr.12	H_Apr.12	N_Apr.12	0.8083	7	2375.6	0.21	0.074
4		C_Mar.14			C_Apr.12	H_Apr.12	N_Apr.12	0.8058	6	2375.7	0.23	0.073
5	C_Feb.15	C_Mar.14	H_Mar.14		C_Apr.12		N_Apr.12	0.808	7	2375.9	0.47	0.065
16		C_Mar.14	H_Mar.14	N_Mar.14	C_Apr.12	H_Apr.12	N_Apr.12	0.809	8	2377.1	1.66	0.036
17	C_Feb.15	C_Mar.14	H_Mar.14			H_Apr.12	N_Apr.12	0.8045	7	2378.6	3.21	0.016
18		C_Mar.14	H_Mar.14			H_Apr.12	N_Apr.12	0.8016	6	2378.9	3.5	0.014
19		C_Mar.14				H_Apr.12	N_Apr.12	0.7983	5	2379.4	4.01	0.011
20	C_Feb.15	C_Mar.14				H_Apr.12	N_Apr.12	0.8	6	2380.2	4.74	0.008

*C, vegetation cover area; H, averaged height; N, averaged normalized difference vegetation index (NDVI) of the sampling points.*

### Comparison Between Manually Sampled and Predicted Values

The set of predicted yields in each plot (excluding the 6 plots used for destructive sampling of soybean plants) was determined by using the GAUSS framework. The manually collected wheat yield of a plot often differed from the set of predicted values for that plot (Figure 4B). The manually collected values fell outside the quartiles of the predicted values in 54.7% of the plots (35 of 64 plots) and most of these (25) were less than the 25% quartile of the overall distribution of predicted values for the plot.

### Differences Among Soybean Cultivars

The measured traits of the soybeans showed considerable variability among cultivars (Supplementary Table 2). Interestingly, conventional statistical analysis of the actual wheat yield data did not identify significant variations among the soybean cultivars in their effect on subsequent wheat yield (ANOVA, *F* = 0.928, *p* = 0.536, Supplementary Figure 2). However, the GAUSS analysis identified significant differences among cultivars in the predicted wheat yields (Figure 4C and Supplementary Table 4), after the effects of spatial variations were removed statistically. Cultivar v5 was associated with the lowest yield in the subsequent wheat crop, and v11 was associated with the highest yield (Figure 4C). There was no significant difference in wheat yields between soybean sowing dates (Supplementary Table 4).

### Soybean Traits Associated With Wheat Yield

**FIGURE 5:**
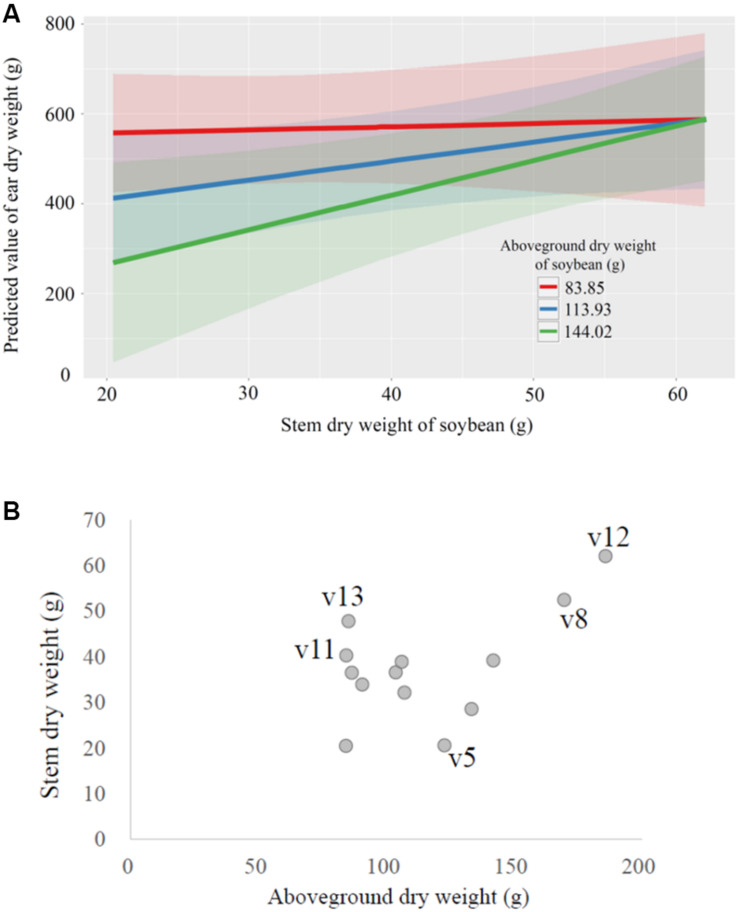
Relationships between selected crop characteristics. **(A)** The marginal effects of the above-ground dry weight and the stem dry weight of soybeans (*Glycine max*) on the predicted ear dry weight of the subsequently grown wheat (*Triticum aestivum*) crop. **(B)** The relationship between above-ground dry weight and stem dry weight of the 14 soybean cultivars.

The interaction of soybean stem dry weight × above-ground dry weight had a significant effect on wheat yield (ear dry weight; *P* < 0.05; Supplementary Table 5). Increased above-ground dry weight of whole soybean plants apparently reduced subsequent wheat yield, but as the stem weight of soybean increased, the wheat yields also increased (Figure 5A). Interestingly, where the above-ground weight of soybean plants was low, the wheat yield was high, regardless of the stem weight of soybean (Figure 5A). In fact, the soybean cultivars associated with low yields in wheat were usually those that had relatively large above-ground weight and relatively small stem weight, and vice versa (Figures 4C, 5B). The 100-seed weight of soybean seeds also significantly affected the wheat yield (*P* < 0.05, Supplementary Table 5 and Supplementary Figure 3). The interactions of aboveground dry weight × seed dry weight and of stem dry weight × seed dry weight did not significantly affect wheat yield (Supplementary Table 5).

## Discussion

This study used a simple analytical framework, identified as GAUSS, to analyze UAV-supported data from field experiments. The performance of this framework was assessed by analyzing data from a crop rotation trial of soybean and wheat. This framework acquired a large amount of high-spatial-resolution data for predicted wheat yield. Analysis of this data showed significant differences among the soybean cultivars in the yield of the wheat grown after them, and identified the soybean traits associated with increased yield. Despite the long history of legume plants in crop rotations (Chorley, 1981; Stinner et al., 1992), to the best of our knowledge this is the first field study that has identified which traits were associated with the benefits of rotation. Although the conventional analysis of manual sampling data did not identify significant differences among soybean cultivars in their effect on wheat yield (Supplementary Figure 2), the GAUSS approach did detect such differences. The large quantity of predicted values with location information generated by this methodology enabled the statistical analysis to include the intra- and inter-plot variations. This suggests that GAUSS has the potential to considerably enhance field experimentation, thereby improving its usefulness.

Soybean cultivars with relatively small above-ground weight, large stem weight, and low 100-seed weight were associated with increased yield in the subsequently grown wheat crop. This suggests several implications for studies of legume-based crop rotations. First, the negative effect of increased above-ground weight of soybean on wheat yield may be attributable to the removal of the above-ground parts of soybean from the field at harvest. Soybean cultivars with large above-ground weight likely absorbed more soil nutrients from the soil than the cultivars that produced small above-ground parts. This removal would have decreased the available nutrient pool for subsequent wheat growth. Second, a study comparing 383 soybean cultivars shows a high correlation between stem and root weight (*r*^2^ = 0.81–0.90; Nakamura and Sawahata, 1988). Therefore the soybean cultivars with large stem weights likely produced large amounts of roots, which may have affected the soil physical and chemical conditions; for example, aggregate structure, release of nitrogen compounds, and biological activities (decomposition) may have been enhanced, increasing the yield of the subsequent wheat crop. However, it should be noted that this experiment was conducted in a single location and a single growing season. Multi-year trials at various locations would be necessary for a more convincing conclusion and a cost-effective GAUSS approach would be useful.

This study did not measure any below-ground soybean traits, such as root biomass or number of nodules. Variations among soybean cultivars in the symbiotic performance of rhizobia are known to occur (Appunu et al., 2008). The variations in the effect of soybean cultivars on wheat yield observed in this experiment may reflect these differences in rhizobial activity. However, preliminary assessments found that the number of rhizobium nodules on the roots of soybeans grown in this field was low. There were no clear differences among the cultivars (data not shown), likely owing to the relatively high soil nutrient content in this field. Future studies are needed to clarify how soybean root residues change soil biophysical properties and increase the yield of subsequent crops. It remains unclear why the 100-seed weight reduced the wheat yield. Chromosome segment substitution lines for 100-seed weight (Liu et al., 2018) may be useful for exploring the causal relationship, but that is beyond the scope of the work reported here. The differences in total seed weight among the soybean cultivars were not associated with differences in wheat yield. This suggests that it may be possible to select soybean cultivars that provide both sufficient soybean yields and crop rotation benefits for the subsequent crop.

In this study, we did not measure the soil nitrate concentration for each rotational treatment. In crop rotations with legumes, the effect of soil nitrogen accumulation on subsequent crops has been reported to be highly variable (Walley et al., 2007; Anglade et al., 2015). One reason for the variation is that non-nitrogen (non-N) factors (such as the bio-physical change in soil properties due to legume residues, other plant nutrients, disease suppression, and weed control) contribute significantly to crop rotational benefits (Stevenson and Van Kessel, 1996; Arcand et al., 2014; Uzoh et al., 2017). In order to verify how the soybean varieties affect the yield of subsequent crops, it will be necessary not only to quantify the change in the soil nitrogen accumulation, but to also examine other bio-physical factors.

The present study applied the GAUSS approach to a crop rotation experiment, but the approach is applicable to a wide range of field experiments. For example, it could be used to study the effects of environmental conditions (water, fertilizer, pesticide, etc.) on yield, with high spatial resolution. GAUSS may also be useful for field experiments in ecology and environmental science. It could be used to measure any plant traits that can be estimated from UAV (or potentially satellite) imagery. For example, UAV imaging and image analysis may enable estimation of important functional traits of complex plant communities, such as biomass, volume, plant height, and photosynthetic activity. The relationship between these functional traits and plant diversity has been examined in grassland field experiments all over the world (Tilman et al., 2006; Cardinale et al., 2007; Zavaleta et al., 2010; Sasaki et al., 2017), but the GAUSS approach has potential to greatly enhance these experiments.

GAUSS also has potential to markedly reduce the time needed for yield surveys, which could facilitate greater numbers of experimental treatments and replicates. For example, in the study reported here, manual collection and measurement of 157 one-square-meter wheat samples required more than 450 person-hours, whereas the UAV drone surveillance, data processing, and GIS analysis took approximately 20 person-hours for GAUSS to estimate the yield of 8,756 cells (total 547.25 m^2^) in the same experiment.

The GAUSS approach worked well here, but there are many limitations and challenges that remain to be addressed to facilitate its extensive adoption for field experimentation. First, although the predictive model based on the UAV imagery was relatively accurate (*r*^2^ = 0.8061), this model was based on a relatively small number of UAV imagery datasets on 3 days (a total of seven variables: two height values, two NDVI values, and three cover area values). Increasing the frequency of UAV sensing and adding more explanatory variables could produce an even better predictive model. The method used here to identify the best model was limited by the number of UAV surveys and the number of explanatory variables. If the frequency of UAV sensing is increased and a large number of explanatory variables is included, more flexible analytical methods, such as machine learning, may be useful for estimating models.

Second, the size of grid cells needs to be optimized. In this study, the GIS grid size (25 cm × 25 cm) was based on the size of an individual wheat plant. However, the grid can be any size, depending on the size and scale of the target species.

Third, the appropriate size for experimental plots needs to be determined. Here, the plot shape and size (2.4 m × 4.2 m) was similar to those in typical field experiments. However, GAUSS can detect differences using smaller plot sizes, which could improve the efficiency of field experiments. Future studies will be needed to identify and validate appropriate plot sizes for UAV-supported field experiments.

Fourth, although it is more than 100 times more efficient per unit area than manual measurement, the GAUSS method always needs manually sampled data from which to develop its predictive models.

Fifth, the GAUSS approach is somewhat expensive because it requires a UAV (drone) with a multispectral camera and RTK-GPS. However, these costs are likely to decrease substantially as the technology develops further and becomes more widely used.

Sixth, although development of relatively good predictive models may help overcome the inherent large variations among manually collected samples, examinations of diverse plant species under various field conditions (e.g., rice, potato, and maize, in uniform vs. non-uniform fields) are needed to investigate the variability of GAUSS data within field experiments.

Seventh, in step 1 of GAUSS, we used commercial photogrammetric software (Pix4Dmapper Pro, Pix4D, Lausanne, Switzerland) to run the 3D reconstruction of the field. In step 2 and 3, we also run the separated scripts to sample the field and calculate phenotypic traits, those require several manual operations, which are time-consuming. In the future, building an automated pipeline (e.g., CIAT Pheno-i, Selvaraj et al., 2020) will allow us to build predictive models more cost-effectively, easily-to-sue, and quickly.

In conclusion, a new analytical framework for UAV-supported field experimentation was proposed. This framework may be applicable to a wide range of field experimentation in crops and wild plants. It could improve the way field experiments are conducted, which has not changed much since Fisher’s era. Wider usage and resolution of the limitations and challenges will likely enable the proposed GAUSS framework to be used consistently in future field experiments.

## Data Availability Statement

The datasets generated for this study are available on request to the corresponding author.

## Author Contributions

YF, WG, SO, NA, SK, and HK conducted field experiment. WG conducted drone sensing and image analysis. All authors discussed, wrote the manuscript, and designed the experiment.

## Conflict of Interest

The authors declare that the research was conducted in the absence of any commercial or financial relationships that could be construed as a potential conflict of interest.

## Supplementary Material

Supplementary Figure 1 The locations of the manual sampling points in the experimental field.

Supplementary Figure 2 Comparison of the effects of different soybean (Glycine max) cultivars grown before the wheat (Triticum aestivum) crop on ear dry weight of wheat.

Supplementary Figure 3 The marginal effect of 100-seed dry weight of soybean (Glycine max) on the predicted values of ear dry weight of subsequently grown wheat (Triticum aestivum).

Supplementary Table 1 Monthly means of mean air temperature, daily solar radiation, and total precipitation.

Supplementary Table 2 Cultivar identities and values of measured traits of the soybeans (Glycine max) grown in this study.

Supplementary Table 3 List of experimental plots.

Supplementary Table 4 Results of generalized linear mixed model (GLMM) analysis of wheat (Triticum aestivum) ear weight in response to previously grown soybean (Glycine max) cultivars.

Supplementary Table 5 Results of generalized linear mixed model (GLMM) analysis of wheat (Triticum aestivum) ear weight in response to specific traits of previously grown soybeans (Glycine max).
