# Supplementary material for: GIS-Based Analysis for UAV-Supported Field Experiments Reveals Soybean Traits Associated With Rotational Benefit
Source: Front Plant Sci. 2021 May 31;12:637694. doi: 10.3389/fpls.2021.637694 (PMC8201397; doi:10.3389/fpls.2021.637694)
Supplement: Supplementary Figure 1 — The locations of the manual sampling points in the experimental field. [file Data_Sheet_1.docx]

**Supplementary Materials**

**Figure S1** Locations of the 154 manual sampling points in the experimental field.

**Figure S2** Comparison of the effects of different soybean (*Glycine max*) cultivars grown before the wheat (*Triticum aestivum*) on actual wheat ear dry weight. “Weedy” indicates plots where weeds were allowed to develop naturally during the soybean production period; “sheet” indicates plots where the soil was covered with an anti-weed sheet during the soybean production period. The black bar within a box indicates the median; box bottom and top, 25 and 75% quartiles, respectively; whiskers, 1.5× the interquartile range.


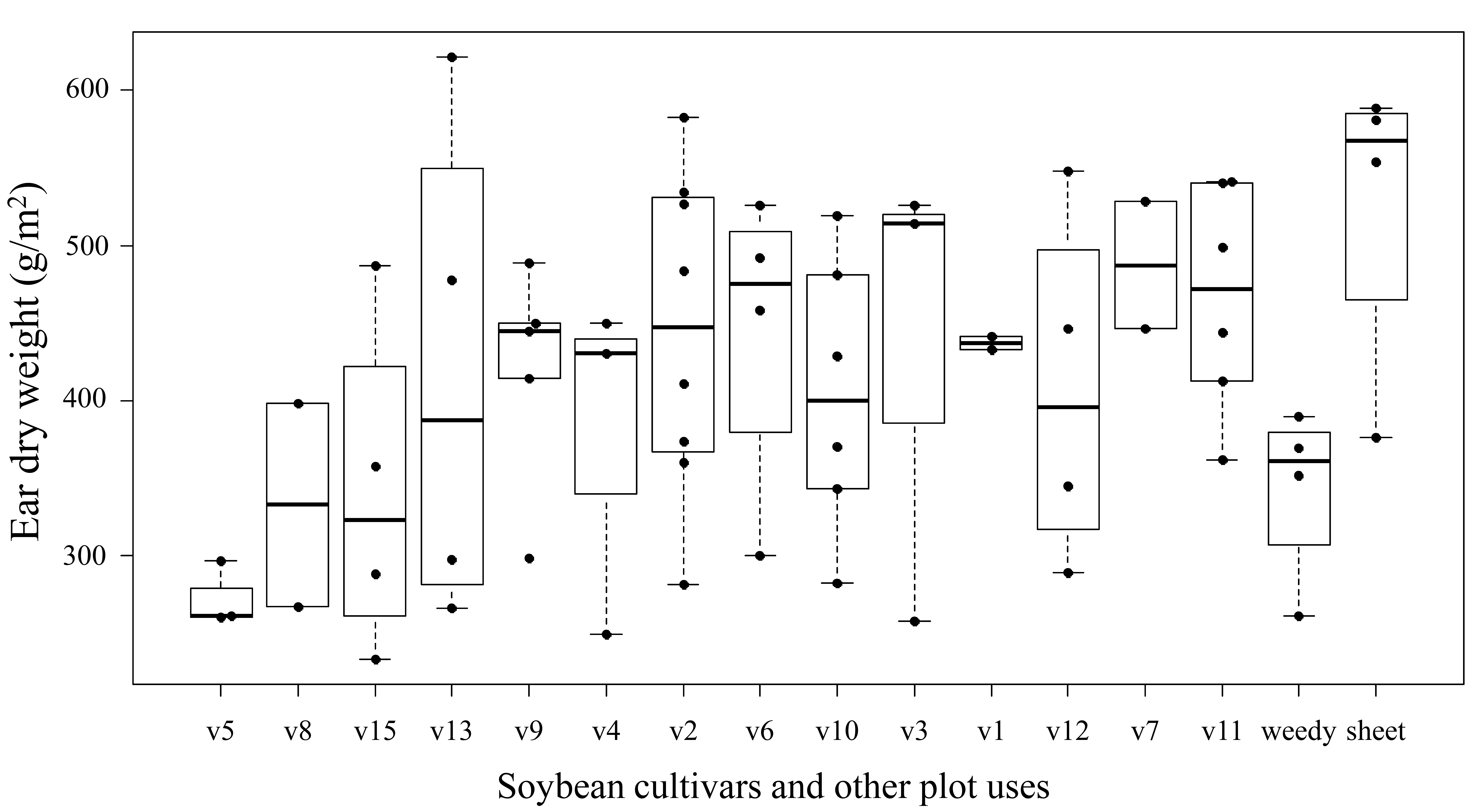


**Figure S3** The marginal effect of the 100-seed dry weight of soybeans (*Glycine max*) on the predicted values of ear dry weight of subsequently grown wheat (*Triticum aestivum*).

**Table S1**. Monthly means of air temperature, daily solar radiation, and total precipitation.

| Date  (month and year) | Mean temperature (°C) | Solar radiation (MJ m^−2^ d^−1^) | Precipitation  (mm) |
| --- | --- | --- | --- |
| Jun 18 | 23.3 | 491.3 | Data not available |
| Jul 18 | 29.1 | 618.4 | Data not available |
| Aug 18 | 28.8 | 565 | Data not available |
| Sep 18 | 23.4 | 309 | Data not available |
| Oct 18 | 19.5 | 339.5 | 66.3 |
| Nov 18 | 14.2 | 266.4 | 26.4 |
| Dec 18 | 8.2 | 234.7 | 44.6 |
| Jan 19 | 5.1 | 313.9 | 14.0 |
| Feb 19 | 7.3 | 294.2 | 37.1 |
| Mar 19 | 10.9 | 441.5 | 128.9 |
| Apr 19 | 14.6 | 545.8 | 91.9 |
| May 19 | Data not available | Data not available | Data not available |
| Jun 19 | Data not available | Data not available | Data not available |

**Table S2** Cultivar identities and values of measured traits of the soybeans (*Glycine max*) grown in this study.

| Cultivar | Field ID | Sample size | Above-ground dry weight (g) | | Stem dry weight  (g) | | Seed dry weight  (g) | | 100 seed dry weight  (g) | | Number of seeds | |
| --- | --- | --- | --- | --- | --- | --- | --- | --- | --- | --- | --- | --- |
|  |  |  | mean, ± standard deviation | | | | | | | | | |
| GmWMC015 | v1 | 2 | 142.100 | ± 8.910 | 39.193 | ± 14.202 | 40.310 | ± 21.298 | 12.315 | ± 0.191 | 326.023 | ± 167.890 |
| GmWMC022 | v2 | 4 | 133.538 | ± 14.436 | 28.538 | ± 10.441 | 52.534 | ± 7.099 | 7.403 | ± 0.531 | 717.003 | ± 147.680 |
| GmWMC024 | v3 | 3 | 103.867 | ± 26.178 | 36.597 | ± 13.825 | 38.885 | ± 7.706 | 11.837 | ± 1.172 | 326.587 | ± 33.743 |
| GmWMC027 | v4 | 4 | 107.263 | ± 18.791 | 32.164 | ± 2.151 | 44.763 | ± 5.567 | 13.923 | ± 0.899 | 322.508 | ± 43.372 |
| GmWMC036 | v5 | 2 | 122.925 | ± 10.642 | 20.603 | ± 5.158 | 49.405 | ± 1.245 | 6.035 | ± 0.488 | 820.489 | ± 45.711 |
| GmWMC094 | v6 | 4 | 106.200 | ± 19.962 | 38.891 | ± 11.649 | 38.480 | ± 9.959 | 22.220 | ± 0.804 | 173.595 | ± 46.264 |
| GmWMC108 | v7 | 2 | 90.758 | ± 61.154 | 33.920 | ± 22.281 | 29.345 | ± 22.111 | 11.370 | ± 1.117 | 249.743 | ± 169.930 |
| Miyagishirome | v8 | 2 | 169.825 | ± 19.339 | 52.468 | ± 0.159 | 63.055 | ± 8.210 | 32.885 | ± 0.431 | 191.924 | ± 27.482 |
| GmWMC150 | v9 | 4 | 86.625 | ± 32.917 | 36.508 | ± 12.406 | 7.848 | ± 2.977 | 4.555 | ± 0.474 | 174.664 | ± 69.975 |
| GmWMC159 | v10 | 4 | 118.088 | ± 30.145 | 44.278 | ± 12.439 | 15.044 | ± 2.222 | 5.473 | ± 0.294 | 276.420 | ± 49.186 |
| GmWMC165 | v11 | 4 | 84.463 | ± 18.946 | 40.289 | ± 9.876 | 21.088 | ± 5.213 | 8.730 | ± 0.599 | 244.692 | ± 72.354 |
| GmWMC176 | v12 | 3 | 186.017 | ± 33.92 | 62.062 | ± 9.157 | 44.750 | ± 12.233 | 15.557 | ± 1.310 | 292.583 | ± 96.020 |
| GmWMC192 | v13 | 4 | 85.338 | ± 22.263 | 47.804 | ± 7.299 | 3.480 | ± 0.410 | 2.660 | ± 0.345 | 132.775 | ± 26.096 |
| Enrei | v15 | 4 | 84.375 | ± 11.496 | 20.485 | ± 10.943 | 35.956 | ± 6.987 | 25.878 | ± 1.057 | 138.561 | ± 23.936 |

**Table S3** List of experimental plots.

| Plot ID | Field ID | Seeding date (month and day) | Seeding row |
| --- | --- | --- | --- |
| 1 | v4 | Jun 20 | 1 |
| 2 | v9 | Jun 20 | 1 |
| 3 | v13 | Jun 20 | 1 |
| 4 | v2 | Jul 20 | 1 |
| 5 | v15 | Jul 20 | 1 |
| 6 | v2 | Jul 20 | 2 |
| 7 | v1 | Jun 20 | 2 |
| 8 | v6 | Jun 20 | 2 |
| 9 | sheet | Jul 20 | 2 |
| 10 | v10 | Jul 20 | 2 |
| 11 | v11 | Jul 20 | 3 |
| 12 | v2 | Jun 20 | 3 |
| 13 | v12 | Jun 20 | 3 |
| 14 | v11 | Jun 20 | 3 |
| 15 | v3 | Jun 20 | 3 |
| 16 | v10 | Jun 20 | 4 |
| 17 | v10 | Jun 20 | 4 |
| 18 | weed | Jul 20 | 4 |
| 19 | v5 | Jun 20 | 4 |
| 20 | v4 | Jun 20 | 4 |
| 21 | v5 | Jul 20 | 5 |
| 22 | v13 | Jun 20 | 5 |
| 23 | sheet | Jul 20 | 5 |
| 24 | v12 | Jun 20 | 5 |
| 25 | v9 | Jun 20 | 5 |
| 26 | v2 | Jun 20 | 6 |
| 27 | v15 | Jul 20 | 6 |
| 28 | weed | Jul 20 | 6 |
| 29 | v11 | Jun 20 | 6 |
| 30 | v6 | Jun 20 | 6 |
| 31 | destructive sampling | Jun 20 | 7 |
| 32 | destructive sampling | Jun 20 | 7 |
| 33 | destructive sampling | Jun 20 | 7 |
| 34 | v15 | Jun 20 | 7 |
| 35 | v7 | Jun 20 | 7 |
| 36 | v5 | Jun 20 | 8 |
| 37 | v3 | Jun 20 | 8 |
| 38 | weed | Jul 20 | 8 |
| 39 | v13 | Jun 20 | 8 |
| 40 | v15 | Jul 20 | 8 |
| 41 | v5 | Jul 20 | 9 |
| 42 | v4 | Jun 20 | 9 |
| 43 | v2 | Jun 20 | 9 |
| 44 | v9 | Jun 20 | 9 |
| 45 | v6 | Jun 20 | 9 |
| 46 | v8 | Jun 20 | 10 |
| 47 | v11 | Jun 20 | 10 |
| 48 | v15 | Jul 20 | 10 |
| 49 | v10 | Jun 20 | 10 |
| 50 | v11 | Jul 20 | 10 |
| 51 | v1 | Jun 20 | 11 |
| 52 | v7 | Jun 20 | 11 |
| 53 | v4 | Jun 20 | 11 |
| 54 | v6 | Jun 20 | 11 |
| 55 | v9 | Jun 20 | 11 |
| 56 | v2 | Jul 20 | 12 |
| 57 | v3 | Jun 20 | 12 |
| 58 | sheet | Jul 20 | 12 |
| 59 | v10 | Jul 20 | 12 |
| 60 | v10 | Jun 20 | 12 |
| 61 | v13 | Jun 20 | 13 |
| 62 | sheet | Jul 20 | 13 |
| 63 | weed | Jul 20 | 13 |
| 64 | v2 | Jul 20 | 13 |
| 65 | v2 | Jun 20 | 13 |
| 66 | destructive sampling | Jun 20 | 14 |
| 67 | destructive sampling | Jun 20 | 14 |
| 68 | destructive sampling | Jun 20 | 14 |
| 69 | v12 | Jun 20 | 14 |
| 70 | v11 | Jun 20 | 14 |

**Table S4** Results of generalized linear mixed model (GLMM) analysis of wheat (*Triticum aestivum*) ear weight in response to previously grown soybean (*Glycine max*) cultivars.

| Response variable | Explanatory variables | χ^2^ | DF | *P*-values |
| --- | --- | --- | --- | --- |
| Predicted values of ear dry weight | sowing date | 1.652 | 2 | 0.438 |
|  | cultivars | 29.19 | 13 | 0.008 |
|  | v5 vs. v8 | 0.35 | 1 | 0.552 |
|  | v5 vs. v15 | 0.47 | 1 | 0.492 |
|  | v5 vs. v13 | 41.08 | 1 | 0.000 |
|  | v5 vs. v9 | 6.37 | 1 | 0.012 |
|  | v5 vs. v4 | 4.49 | 1 | 0.034 |
|  | v5 vs. v2 | 2.54 | 1 | 0.111 |
|  | v5 vs. v6 | 5.26 | 1 | 0.022 |
|  | v5 vs. v10 | 8.423 | 1 | 0.004 |
|  | v5 vs. v3 | 2.32 | 1 | 0.128 |
|  | v5 vs. v1 | 13.5 | 1 | 0.000 |
|  | v5 vs. v12 | 8.492 | 1 | 0.004 |
|  | v5 vs. v7 | 3.24 | 1 | 0.072 |
|  | v5 vs. v11 | 22.36 | 1 | 0.000 |
|  | v5 vs. weed | 21.98 | 1 | 0.000 |
|  | v5 vs. sheet | 10.70 | 1 | 0.001 |

Cultivar identifiers are as listed in Table S2.

**Table S5**. Results of generalized linear mixed model (GLMM) analysis of wheat (*Triticum aestivum*) ear weight in response to specific traits of previously grown soybean (*Glycine max*).

| Response variable | Explanatory variables | χ^2^ | DF | *P*-values |
| --- | --- | --- | --- | --- |
| Predicted values of ear dry weight | aboveground weight | 1.605 | 1 | 0.20517 |
|  | stem weight | 3.454 | 1 | 0.06308 |
|  | seed weight | 2.083 | 1 | 0.14893 |
|  | 100-seed weight | 5.089 | 1 | 0.02408 |
|  | aboveground weight × stem weight | 4.977 | 1 | 0.02569 |
|  | seed weight × stem weight | 3.152 | 1 | 0.07585 |
|  | aboveground weight × seed weight | 0.293 | 1 | 0.58826 |
